# *ScaleSC*: a superfast and scalable single-cell RNA-seq data analysis pipeline powered by GPU

**DOI:** 10.1093/bioadv/vbaf167

**Published:** 2025-07-17

**Authors:** Wenxing Hu, Haotian Zhang, Yu H Sun, Shaolong Cao, Jake Gagnon, Yuka Moroishi, Yirui Chen, Zhengyu Ouyang, Baohong Zhang

**Affiliations:** Research Department, Biogen, Inc., Cambridge, MA 02142, United States; Research Department, Biogen, Inc., Cambridge, MA 02142, United States; Research Department, Biogen, Inc., Cambridge, MA 02142, United States; Research Department, Biogen, Inc., Cambridge, MA 02142, United States; Research Department, Biogen, Inc., Cambridge, MA 02142, United States; Research Department, Biogen, Inc., Cambridge, MA 02142, United States; Research Department, Biogen, Inc., Cambridge, MA 02142, United States; Data Science, BioInfoRx, Inc., Madison, WI 53719, United States; Research Department, Biogen, Inc., Cambridge, MA 02142, United States

## Abstract

**Summary:**

The rise of large-scale single-cell RNA-seq data has introduced challenges in data processing due to its slow speed. Leveraging advancements in Graphics Processing Unit (GPU) computing ecosystems, such as *CuPy* and Compute Unified Device Architecture (CUDA), building on *Scanpy* and *Rapids-singlecell* package, we developed *ScaleSC*, a GPU-accelerated solution for large-scale single-cell data processing. *ScaleSC* delivers over a 20× speedup through GPU computing and significantly improves scalability, handling datasets of 10–20 million cells with over 1000 batches by overcoming the memory bottleneck on a single A100 card, which far surpasses *Rapids-singlecell’*s capacity of processing only 1 million cells without multi-GPU support. We also resolved discrepancies between GPU and Central Processing Unit (CPU) algorithm implementations to ensure consistency. In addition to core optimizations, we developed novel tools for marker gene identification and cluster merging with GPU-optimized implementations seamlessly integrated. *ScaleSC* shares a similar syntax with *Scanpy*, which helps lower the learning curve for users already familiar with *Scanpy* workflows.

**Availability and implementation:**

The *ScaleSC* package (https://github.com/interactivereport/ScaleSC) promises significant benefits for the single-cell RNA-seq computational community.

## 1 Introduction

Recent advances of single-cell RNA sequencing (scRNA-seq) technology enable researchers to study the gene expression profiles of individual cells. Unlike bulk RNA sequencing, which averages gene expression across a population of cells, scRNA-seq captures the heterogeneity within a cell population, offering unprecedented insights into cell types, states, and dynamic biological processes.

In recent years, pre-trained foundation models based on transcriptomic data, such as scGPT (Cui *et al.* 2024), scFoundation (Hao *et al.* 2024a), and GeneFormer (Theodoris *et al.* 2023), have gained significant popularity. These models are typically trained unsupervised on large-scale single-cell RNA-seq datasets comprising millions of cells. Their primary goal is to predict the effects of gene regulation or drug treatments on gene expression profiles, thereby opening avenues in understanding biological mechanisms and potentially treating disease.

Concurrently, a novel and rapidly growing area of research involves AI-powered virtual cell models (Istrate *et al.* 2024), which aim to simulate cellular behavior. Building such models requires vast, diverse, and multimodal biological datasets. As a result, the ability to preprocess datasets containing millions of cells efficiently is becoming increasingly critical.

scRNA-seq datasets are highly dimensional, encompassing both numerous observations, such as millions of cells, and a vast array of features, such as 30 000 genes. Analyzing scRNA-seq data is a complex, multi-step process aimed at extracting meaningful biological insights from the transcriptomes of individual cells while removing unwanted noise and batch effects. The data analytical workflow includes quality control (QC), normalization, dimensionality reduction, batch effect correction, clustering, cell type annotation, and various downstream analyses. Preprocessing helps mitigate unwanted noise and extract biologically relevant signals from cleaned cells and cell types.

The generated scRNA-seq data, typically delivered as a cell-by-gene matrix, is massive due to the high dimensionality of 20–50k genes and millions of cells. This makes the analytical steps computationally intensive in terms of both space (memory) and time cost. As a result, analyzing large-scale scRNA-seq datasets requires high-performance servers with a huge memory capacity and is time-consuming. To address these computational challenges, specialized tools have been developed to manage the scalability of the data and reduce computational costs. These tools focus on reducing memory usage, parallelizing workflows, and optimizing algorithms for efficient, high-throughput analysis. For example, *HDF5* (https://www.hdfgroup.org/solutions/hdf5/), a dedicated package for fast and flexible I/O for large and complex data (Folk *et al.* 2011), is used to support hierarchical metadata structures and out-of-memory operations. *Anndata* (Virshup *et al.* 2021) is a popular tool designed to effectively manage large cell×gene matrices. Both “in memory” and “on disk” data loading are supported in *Anndata*. *SciPy* (Virtanen *et al.* 2020) supports sparse matrices to further reduce memory storage and speed up computing operations, by leveraging the high sparsity of cell×gene matrices. Highly variable genes (HVG) (Zheng *et al.* 2017, Stuart *et al.* 2019) are used to select features for dimension reduction, reducing from 20–50k features to 1–5k, by excluding genes with small variations. Principal component analysis (PCA) is used subsequently to further reduce feature dimension from 1k–5k to 20–200.

*Scanpy* (Wolf *et al.* 2018), https://scanpy.readthedocs.io/en/stable/, built on *Anndata*, is a one-stop package for single-cell data analysis, including preprocessing, cell clustering, data visualization, etc. *Scanpy* utilizes the efficient methods and data structures aforementioned, and has incorporated other essential scRNA-seq data processing steps, e.g. normalization, quality control, batch effect removal, and clustering. With these features, *Scanpy* has become one of the most popular packages for scRNA-seq data analysis.

Though the use of efficient methods, algorithms, and engineering, data analysis with *Scanpy* remains slow due to the extremely high dimensionality of single-cell RNA sequencing (scRNA-seq) data. Since scRNA-seq data is typically stored in matrix format, a natural approach is to leverage GPUs for parallel computing, as a single GPU card contains thousands of computational units, or CUDA cores. To address this, the scverse core team developed *Rapids-singlecell* (Dicks *et al.* 2024), a GPU-accelerated scRNA-seq analysis package that improves performance by more than 20 times compared to *Scanpy*. While *Rapids-singlecell* was initially limited to datasets with fewer than 1 million cells, recent updates have expanded its capabilities to support massive-scale datasets using multiple GPUs via *Dask*, as well as out-of-core execution. This allows *Rapids-singlecell* to efficiently handle extremely large datasets, beyond the constraints of GPU memory. For instance, PCA on 10 million cells can now be completed in under 10 s on a DGX system i.e. designed by NVIDIA specifically for deep learning and artificial intelligence (AI) workloads.

Despite its improvements, *Rapids-singlecell* still has limitations. While it supports multi-GPU configurations with *Dask* arrays for large datasets, it remains inaccessible to independent researchers who typically lack multiple GPUs or large-scale computing resources. Additionally, its out-of-core computing support, though promising, often faces issues with the *RAPIDS* Memory Manager (RMM), resulting in crashes or stalls. Furthermore, the Harmony algorithm, crucial for batch correction, suffers from high memory consumption and lacks multi-GPU support, limiting its scalability for datasets with many batches.

These limitations impact *Rapids-singlecell’*s scalability, particularly for researchers with constrained resources or datasets that require memory-intensive algorithms like Harmony.

In this work, we introduce *ScaleSC*, a GPU-accelerated package developed on top of *Rapids-singlecell* and *Scanpy*, designed to deliver their superior performance to resource-limited users. *ScaleSC* enables the analysis of large datasets on a single GPU, overcoming the memory constraints of *Rapids-singlecell*. It delivers 20–100 times faster performance than *Scanpy* and can efficiently handle datasets with up to 10–20 million cells (depends on the number of genes). Additionally, *ScaleSC* offers exceptional capabilities in marker gene identification and cluster merging. A more detailed explanation of *ScaleSC* can be found in the Section 2.

## 2 Methods

Building on the foundation of *Rapids-singlecell*, which already implemented most of the essential scRNA-seq data processing steps in the GPU version, we developed *ScaleSC* with several improvements, including (i) fixing the discrepancy of results between *Scanpy* and *Rapids-singlecell*, (ii) solving the scalability bottlenecks, and (iii) optimizing algorithms for memory efficiency.

### 2.1 Discrepancy removal

The output results are different between *Scanpy* and *Rapids-singlecell*. *Scanpy* uses CPU-based scientific computing libraries like *NumPy*, *SciPy*, and *Sklearn*, while *Rapids-singlecell* is mainly based on the GPU-accelerated *Rapids* AI ecosystem. *Scanpy* and *Rapids-singlecell* implement the same algorithm slightly differently in order to optimize the different computational architectures between CPU and GPU. We refer to this difference as “system variance.” In addition to system variance, floating-point errors also contribute to differences in results, and we refer to this as “numerical variance.” Although neither “system variance” nor “numerical variance” is incorrect, the difference in results leads to inconsistencies in data processing, posing challenges for reproducibility. Numerical variance is unavoidable but less significant compared to system variance and can be mitigated by increasing precision settings. This section focuses primarily on how *ScaleSC* eliminates system variance.

#### 2.1.1 Principal component analysis discrepancy

Principal component analysis is a critical step in single-cell RNA-seq pipeline, which efficiently projects high-dimensional single-cell count data into low-dimensional space for conducting downstream analysis. Principal component analysis typically consists of two steps:

- **Standardization**: The count value of each gene in each cell $X_{ij}$ is normalized to a mean of 0 and a deviation of 1 as follows,
  $$Z_{ij}=\frac{X_{ij}-\mu_{i}}{\sigma_{i}+\epsilon }$$
   where $Z_{ij}$ is the normalized count of gene *i* in cell *j*, $\mu_{i}$ is the mean count of gene *i* over all cells, $\sigma_{i}$ is the standard deviation of gene *i* over all cells, ϵ is a constant to avoid divided by zero.
- **Eigen-Decomposition**: An Eigen-Decomposition is performed on the covariance matrix. The eigenvectors represent the directions (principal components), and the eigenvalues represent the magnitude of the variance along those directions.
  $$Z^{T}Zv_{i}=\lambda_{i}v_{i}$$

   where $v_{i}$ is the *i*-th principal component and $\lambda_{i}$ is the corresponding eigenvalue of the *i*-th eigenvector.

The differences are raised by the sign of eigenvectors i.e. not uniquely defined, flipping the signs does not affect their mathematical validity, but this can result in a different visualization and make the following analysis not reproducible, e.g. batch removal algorithm Harmony (Korsunsky *et al.* 2019) takes principal components as inputs. As what we found, *Scanpy* internally calls PCA function from *Sklearn*, which aligns the direction by flipping the sign of $v_{i}$ such that the largest absolute value in $v_{i}$ is always positive. The correction can be formulated as below.


$$\tilde{v_{i}}=\mathrm{sign}(v_{ik})v_{i}$$

where *k* is the index of the largest absolute value. Instead of calling PCA from *Sklearn*, *Rapids-singlecell* relies on *RAPIDS* suite that doesn’t correct it automatically, which makes the discrepancy appear. To further address this issue, one more step for correction of principal components is introduced in *ScaleSC*.

#### 2.1.2 Harmony discrepancy

Harmony is a powerful method designed to integrate single-cell datasets from multiple samples or experiments while preserving the biological variation and removing batch effects. It operates on low-dimensional embeddings, typically generated by PCA, then initializes clusters by K-Means based on embeddings and repeatedly adjusts them until convergence.

We found that the discrepancy arises from K-Means clustering and randomness caused by Random Number Generator (RNG), even when the random seed remains unchanged. More specifically, CPU typically generates random numbers sequentially using algorithms like Linear Congruential Generators (LCG), and is deterministic when initialized with the same seed, while GPU generates random numbers in parallel without any guarantee of ordering. This property leads to the uncertainty of algorithms involving randomness, like K-Means. To ensure *ScaleSC* obtains the same initial clusters, the starting points of K-Means need to be matched up. We modify the original Python implementation *harmonypy* in two ways. The first approach is replacing GPU implementation of K-Means with CPU implementation entirely, which minimizes the code changes, and we call it 1-step modification. The second approach is switching the calculation of starting centroids to CPU, then feed them to GPU-accelerated K-Means, we found this way can maximally leverage GPU capacity compared to “1-step” modification, and this refers to 2-step modification.

#### 2.1.3 Neighbor discrepancy

Finding the nearest neighbors based on the Harmony outputs is essential for subsequent Leiden clustering. We aim to construct clusters using the same neighborhood graph and cell connectivities. However, we observe that *Scanpy* and *Rapids-singlecell* generate two distinct graphs from the same input. Upon a detailed review of both *Scanpy* and *Rapids-singlecell* implementations, we identified differences in their nearest neighbor algorithms. *Rapids-singlecell* utilizes an exact k-Nearest Neighbor (k-NN) algorithm, implemented in parallel via *cuML*, while *Scanpy* employs an approximate k-NN method using NNdescent (Dong *et al.* 2011), an algorithm designed for approximating nearest neighbor searches in high-dimensional spaces. To validate this distinction, we force *Scanpy* to use the exact k-NN algorithm and confirmed that the results aligned with those produced by *Rapids-singlecell*. Consequently, *ScaleSC* will call *Rapids-singlecell* internally, using exact k-NN rather than the approximation.

By incorporating the improvements described above, *ScaleSC* can reproduce *Scanpy’*s results in most steps. However, even with these discrepancies resolved, certain steps involving stochastic algorithms, such as Leiden, Uniform Manifold Approximation and Projection (UMAP), and t-distributed Stochastic Neighbor Embedding (t-SNE), may not consistently yield identical results. These variations, primarily introduced during visualization, do not impact the conclusions of quantitative analyses.

### 2.2 Scalability

Frequent interaction with the count matrix can’t be avoided in the single-cell RNA-seq preprocessing pipeline, and loading the whole matrix into memory during runtime is infeasible. Due to its high sparsity (typically >90% non-zero values), equivalent sparse formats are preferred for storage and computing on both CPU and GPU. However, there are two bottlenecks that prevent us from actually using GPU:

- **VRAM Bottleneck**: Most of high-end GPUs in the market have limited memory. Even like the most advanced NVIDIA A100 has only 80*G*, which is insufficient for one-time loading for dataset even with sparse matrix supported.
- **Indexing Bottleneck**: *CuPy* as the backend of sparse matrix on GPU, one notable limitation is the integer type used for indexing, which is fixed as 32-bit integers, indicating it can only store non-zero elements up to $2^{31}-1$, or ∼2.1 billion. This is not enough for extremely large single-cell dataset. For example, a dataset with $5\times 10^{6}$ cells, $2\times 10^{4}$ genes, and a sparsity of 0.9, the number of non-zero elements is much larger than that upper limit. Currently, there is no solution to solve it directly.

To work around with extremely large dataset, we adopt the chunking approach to split the dataset into chunks along cells, so that each data chunk won’t reach the Int32 bottleneck. In addition, this also gives us flexibility to process data chunks separately across devices, which indicates there is a potential way to process arbitrarily large data.

#### 2.2.1 *ScaleSC* overview

**Figure 1.**
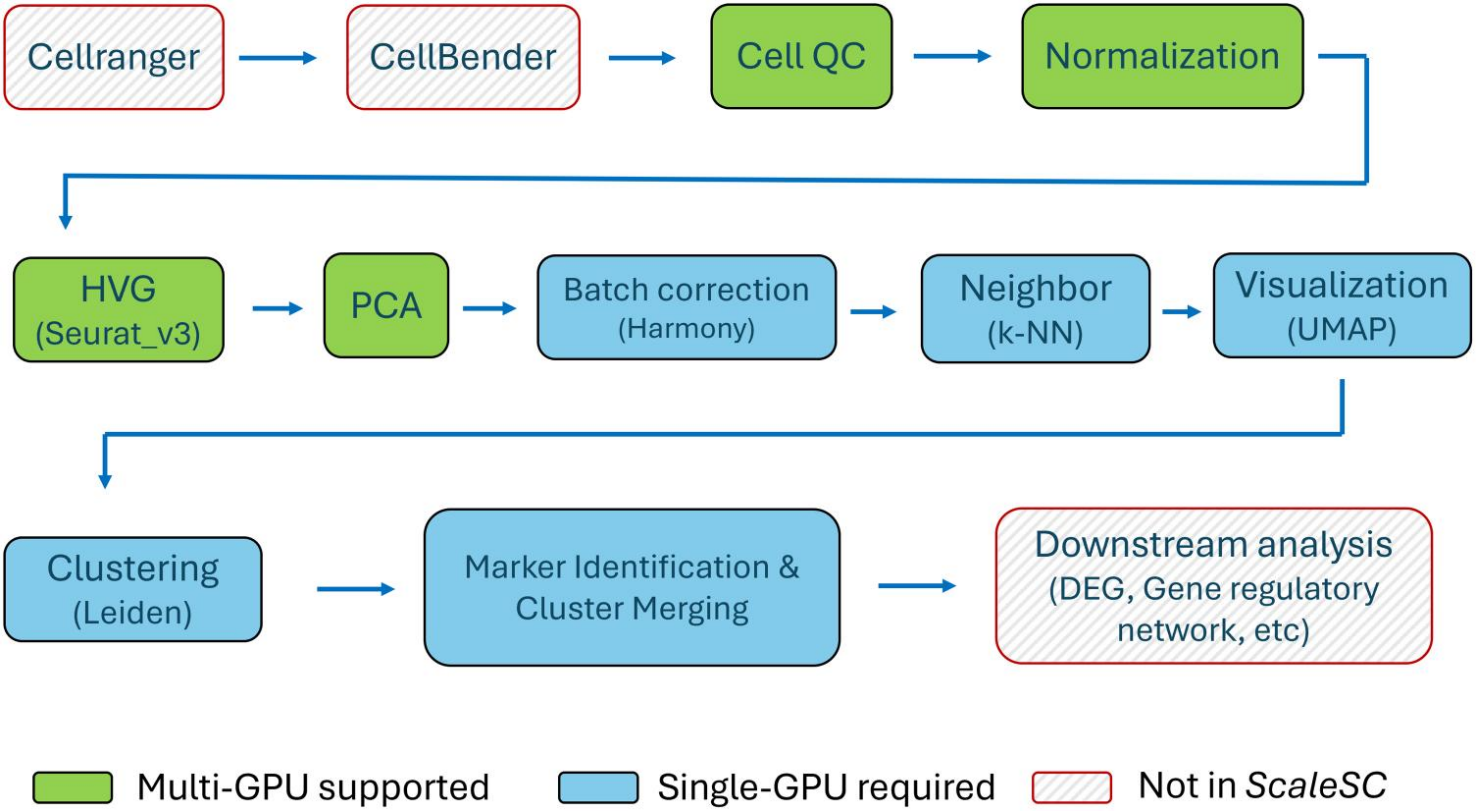
The workflow of standard scRNA-seq data analysis.

Next, we build *ScaleSC* on top of *Scanpy* and *Rapids-singlecell* so that our method can seamlessly scale to tens of millions of cells with GPU acceleration. Like *Scanpy* and *Rapids-singlecell*, *ScaleSC* implements a series of necessary methods in the classical single-cell RNA-seq pipeline, including steps shown in Fig. 1. A comprehensive stepwise illustration is provided in Table 2, available as supplementary data at *Bioinformatics Advances* online.

**Table 2.** The time cost comparison between *Scanpy* and *ScaleSC* on the 1.3M mouse brain data (Zheng *et al.* 2017)^a^.

Step	*Scanpy* (s)	*ScaleSC* (s)
Data loading	26.0	16.1
QC	271.6	4.9
Normalization	21.3	2.0
HVG	312.5	8.5
PCA	269.6	21.7
Harmony	8818.1	23.5
Neighbors	336.5	34.1
UMAP	2404.3	4.4
Leiden clustering	3793.2	10.9
**All**	**16 253.1**	**126.0**

a *Seurat* fails to load the data; *Rapids-singlecell* is not compared here due to *CuPy’*s indexing limitation.

A bold value is the sum of time cost for each algorithm.

1. Cell QC: Filter out low-quality cells and genes according to the number of counts.
2. Normalization: Perform log1p normalization on counts by log(1+X), where *X* is the count value.
3. HVG: Choose highly variable genes using *Seurat* v3 (Stuart *et al.* 2019).
4. PCA: Dimensionality reduction using PCA.
5. Batch Correction: Invoke Harmony to remove batch effects.
6. Neighbor: Use *k*-NN to find the *k* nearest neighbors to build the neighborhood graph.
7. Visualization: Call UMAP on PCA-reduced matrix for better visualization.
8. Clustering: Call Leiden clustering.
9. Marker Identification: Identify cluster-specific genes.
10. Cluster Merging: Clusters are merged to eliminate those that are deemed non-informative, based on the markers detected during the analysis.

**Figure 2.**
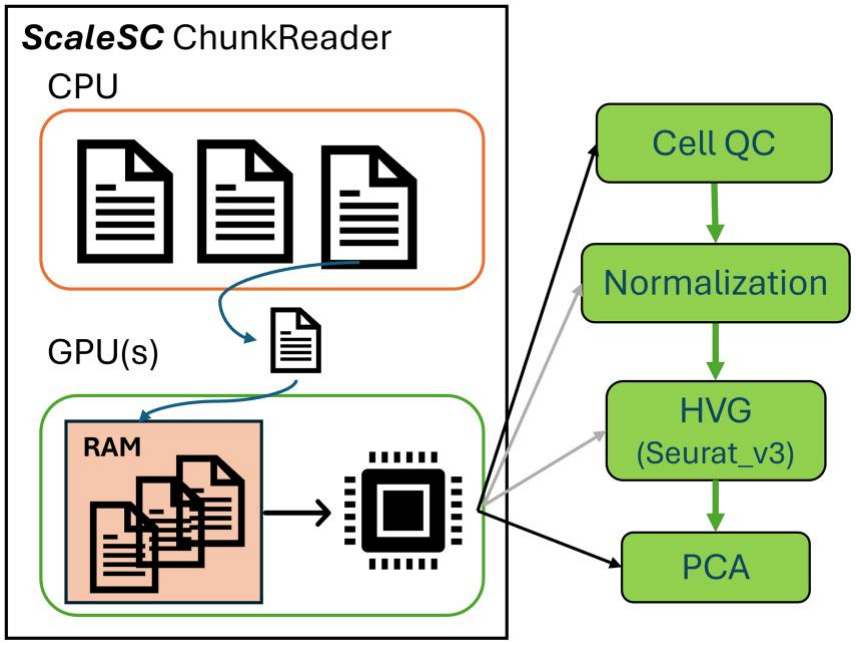
The flowchart chunk-based data loading and preprocessing.

*ScaleSC* proceeds with data loading and preprocessing in chunks, we design a chunked-data reader, as shown in Fig. 2, dedicated to chunked data loading and computation. This reader supports three different modes for loading data under various scenarios, depending on the user’s computing resources (GPU and CPU capability). The three data loading modes are described in the next subsections.

#### 2.2.2 Time-efficient but high memory consumption

In the implemented architecture, *ScaleSC* employs an optimized memory management strategy by pre-loading all data chunks into GPU memory (VRAM). The processing pipeline operates iteratively, where individual chunks are sequentially processed, followed by automatic result aggregation. This approach eliminates the computational overhead associated with CPU-GPU data transfer during processing, as all data is stored in VRAM, thereby maximizing computational efficiency. However, this implementation is constrained by available VRAM capacity, limiting the maximum processable dataset size. To overcome this limitation and accommodate larger datasets, we introduce multi-GPU support for distributed chunk storage. This extension leverages the high-bandwidth of GPU-to-GPU data transfer capabilities through NVSwitch, which significantly outperforms CPU-to-GPU transfer rates. Notably, while data chunks are distributed across multiple GPUs for storage, computation remains centralized on a single GPU to maintain processing efficiency and algorithmic consistency.

#### 2.2.3 Balanced on both time and memory

In contrast to the GPU-centric approach, *ScaleSC* implements an alternative mode where data chunks are pre-loaded and maintained in CPU memory rather than GPU VRAM. In this implementation, the processing pipeline transfers individual chunks sequentially to GPU for computation, followed by result retrieval back to the CPU memory. While this approach introduces additional overhead from CPU-GPU communication, it circumvents GPU memory constraints by leveraging typically larger CPU memory capacity. This implementation’s performance is primarily bounded by available CPU memory and includes latency from host-device communication overhead. However, empirical evidence suggests this mode provides robust performance across a wide range of real-world applications, offering a practical balance between computational efficiency and memory consumption.

#### 2.2.4 Memory efficient but time-consuming

For resource-limited users, *ScaleSC* implements a disk-based processing mode that minimizes memory requirements. In this implementation, data chunks are loaded directly from disk storage on demand, rather than being pre-loaded into either CPU or GPU memory. While this approach introduces significant input/output overhead between disk and host memory, it provides a minimal memory footprint at the cost of increased processing time. This mode’s primary advantage lies in its theoretical capacity to process datasets of arbitrary size, constrained only by available disk space rather than system memory. The trade-off between processing speed and memory utilization makes this implementation particularly valuable for environments with limited computational resources, ensuring *ScaleSC*’s accessibility across diverse computational infrastructures.

### 2.3 Optimizations in *ScaleSC*

This section examines the architectural modifications implemented in *ScaleSC* to efficiently process chunked data structures. We detail the algorithmic adaptations necessary for chunked-based computation and subsequently explore enhancements to the batch correction algorithm Harmony to accommodate substantially larger sample sizes. These modifications are essential for processing large-scale single-cell datasets combined from many experiments.

#### 2.3.1 Cell QC

Within each individual chunk, a subset of cells is retained, while all genes are preserved. This approach facilitates the filtering of cells based on the number of gene counts. In contrast, selecting genes based on the number of cells presents a challenge, as other chunks are not accessible when processing a specific chunk. To address this, an array is maintained to track the number of cells associated with each gene, which is updated incrementally as new chunks are processed. Ultimately, a finalized list of cells and genes is generated following a single iteration over all chunks. This procedure requires traversing all chunks once to ensure the completeness of the filtering process.

#### 2.3.2 Highly variable genes

This section outlines the implementation of the HVG selection step, following the methodology described in the *Seurat* v3 paper (Stuart *et al.* 2019). The workflow can be summarized as follows: *N* represents the number of cells and *M* represents the number of genes:

1. The means and variances for each gene are computed using the raw count matrix. This process requires a single iteration over all data chunks to calculate the means, denoted as $\mu_{i}$, for the *i*th gene, and the sample variances, denoted as $\sigma_{i}$, for the *i*th gene (using N−1 as the divisor). It is important to note that using population variance instead of sample variance will introduce discrepancies.
  $$\begin{array}{cc} s_{i,k}= & s_{i,k-1}+\sum_{x\in X_{k,i}}x\\ s_{i,k}^{2}= & s_{i,k-1}^{2}+\sum_{x\in X_{k,i}}x^{2}\\ \mu_{i}= & \frac{s_{i,K}}{N}\\ \sigma_{i}= & \frac{N}{N-1}(\frac{s_{i,K}^{2}}{N}-\mu_{i}^{2}) \end{array}$$
    where *K* is number of chunks, $X_{k,i}$ is the *i*th gene column in the *k*-th chunk, *x* is the count value, $s_{i,k}$ is sum of count values in the first *k* chunks, $s_{i,k}^{2}$ is the sum of squared count values in the first *k* chunks.
2. Loess Regression in Logarithmic Space: The locally weighted regression (LOESS) is performed by using the logarithm of means as the predictor and the logarithm of variances as the response variable. While a GPU implementation is not currently available, the method is sufficiently fast and efficient when executed on the CPU.
3. Normalization and Clipping: Normalize the raw counts using the fitted standard deviation and means for each gene, and clip any values exceeding $\sqrt{N}$.
  $$x^{\prime }=\min(\frac{x-\mu }{\sigma^{\prime }},\sqrt{N})$$
    where $x^{\prime }$ is the normalized value, μ is the mean of the gene *i* before normalization, σ is the fitted standard deviation in Step (2).
4. Repeat Step (1) to recalculate the variance of normalized data, then mark genes with the K-largest variances as HVGs.

In HVG, two loops of all chunks are needed: the first loop obtains mean and variance of raw data; the second loop normalizes data with regressed variances.

#### 2.3.3 Principal component analysis

Given the chunked nature of the data, obtaining eigenvectors through Singular Value Decomposition (SVD) is not straight-forward. However, it is considerably easier to derive them through the use of a zero-centered covariance matrix.


$${\left(X-m\right)}^{T}(X-m)=X^{T}X-m^{T}s-s^{T}m+Nm^{T}m$$

where $X\in R^{N\times M}$ represents the entire matrix, $m\in R^{M}$ is a vector of means, $s\in R^{M}$ is a vector representing the sum of count values. We can calculate $X^{T}X$ by simply adding up all chunks.


$$X^{T}X=\sum_{k=1}^{K}X_{k}^{T}X_{k}$$

where $X_{k}$ is the *k*-th chunk, and $X^{T}=[X_{1}^{T},X_{2}^{T},\ldots ,X_{K}^{T}]$. Based on the calculated zero-centered covariance matrix, the principal components can be easily obtained through eigen-decomposition. Similar to the High Variance Genes (HVG) step, *ScaleSC* processes all chunks twice.

#### 2.3.4 Batch correction

Harmony (Korsunsky *et al.* 2019) is a widely used algorithm for batch effects removal. However, during these iterative calculations, the algorithm generates and stores numerous intermediate variables, leading to substantial memory consumption. For datasets with ∼13 million cells and over 1000 samples, the original Harmony implementation encounters significant memory issues, often resulting in out-of-memory (OOM) errors. The memory requirement for such datasets exceeds 220 GB, highlighting a critical limitation in its scalability for large-scale single-cell analyses.

From Table 1, available as supplementary data at *Bioinformatics Advances* online, the obvious bottleneck is *B*, the number of samples. We observe that when *B* increases, it becomes impossible to hold even such a single large matrix under 32-bit float. However, Φ, $\Phi_{\mathrm{moe}}$ and $\Phi_{Rk}$ are extremely sparse containing about *N* non-zero values, where *N* is the number of cells. If we convert them to sparse matrices, a significant amount of memory can be saved. Based on this strategy, a (B,N) dense matrix can be converted to a sparse matrix in only O(N) space (∼0.2G). All algebraic operations involving converting matrices need to be adjusted accordingly as well.

**Table 1.** The time cost comparison between *Scanpy*, *Seurat*, *Rapids-singlecell*, and *ScaleSC* on the 70K human lung data (Travaglini *et al.* 2020).

Step	*Scanpy* (s)	*Seurat* (s)	*Rapids-singlecell* (s)	*ScaleSC* (s)
Data loading	0.9	54.2	0.9	1.4
QC	7.5	7.9	0.1	0.1
Normalization	1.1	6.0	0.0	0.1
HVG	10.5	10.2	0.3	0.2
PCA	14.8	39.4	0.3	0.8
Harmony	131.4	62.7	7.8	22.0
Neighbors	153.5	12.2	0.5	0.7
UMAP	87.5	73.1	0.2	0.2
Leiden clustering	16.2	17.7	2.7	2.0
**All**	**423.4**	**283.4**	**12.8**	**27.5**

A bold value is the sum of time cost for each algorithm.

The original script also used dummy variables to create the matrix Φ, which takes a lot of memory on CPU as well. To further optimize memory performance, we generate a sparse dummy matrix directly instead of a huge dense matrix. Besides those sparse matrices, the original script keeps some unused matrices in memory. We removed them and further optimized the script to save more memory.

After conversion and cleaning, it still fails on 13*M* data. The reason is Harmony produces several extremely large intermediate values. For example, it estimates MoE model parameters as follows:


$$W={\left(\Phi_{Rk}\Phi_{\mathrm{moe}}^{T}+\lambda \right)}^{-1}\cdot \Phi_{Rk}\cdot Z_{\mathrm{orig}}^{T}$$

A detailed explanation of $\Phi_{Rk}$, $\Phi_{\mathrm{moe}}$, and $Z_{\mathrm{orig}}$ is provided in the Table S1, available as supplementary data at *Bioinformatics Advances* online. Multiplication of ${\left(\Phi_{Rk}\Phi_{\mathrm{moe}}^{T}+\lambda \right)}^{-1}$ and $\Phi_{Rk}$ results in an intermediate matrix with shape as (B,N), which is ∼64*G* memory space allocated by GPU for temporary storage. A simple trick using associative property of multiplication can be observed to avoid such large temporary storage appeared during calculations: $\Phi_{Rk}\cdot Z_{\mathrm{orig}}^{T}$ won’t take too much space so that can be computed first, then multiply by ${\left(\Phi_{Rk}\Phi_{\mathrm{moe}}^{T}+\lambda \right)}^{-1}$. To further reduce the memory consumption, we implement a few in-place operators using CUDA to obtain the final result without producing any intermediate results.

### 2.4 Marker gene identification

**Figure 3.**
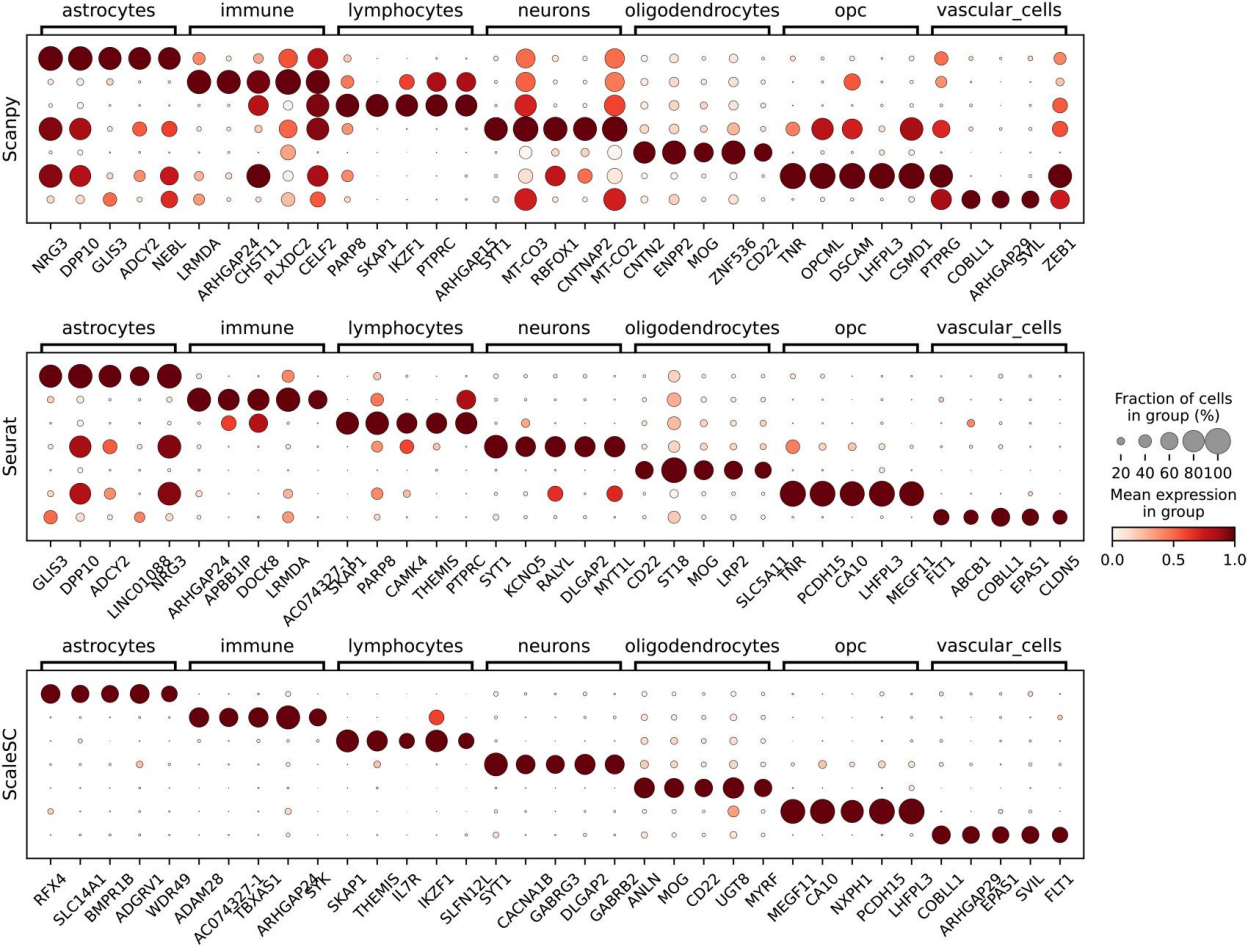
Expression of top-5 gene markers from *Scanpy*, *Seurat*, and *ScaleSC* are compared using a public dataset with 0.06M cells with seven predefined cell types. The three subfigures compare the marker genes identified by *Scanpy*, *Seurat*, and *ScaleSC*, respectively. Columns correspond to the markers, and rows represent the clusters. Brackets indicate cluster-specific markers within each cluster. Larger circles denoting a higher proportion of the marker within the cluster. The expression values are standardized to a range between 0 and 1.

Cell type annotation plays a crucial role in cell type decomposition analysis and understanding biological processes, such as pathways enriched in certain cell types. However, accurately annotating cell types remains a challenge. There are two main approaches: de novo annotation, which relies on marker genes, and reference mapping, which transfers annotations from well-annotated datasets. Marker genes are those that are highly expressed in a specific cluster while showing low expression in others. In traditional pipelines such as *Scanpy* and *Seurat*, marker genes are typically identified using classical statistical methods (Pullin and McCarthy 2024) (e.g. Welch’s *t*-test or Wilcoxon rank-sum test) or simple classifiers such as logistic regression (Ntranos *et al.* 2019). However, the identified markers often lack accuracy and specificity (see Fig. 3).

*NSForest* (Liu *et al.* 2024) presents an advanced methodology by integrating a random forest classifier with network structure analysis. While it offers notable advantages, its high computational complexity has constrained its applicability to even medium-sized datasets, making it impractical for large-scale single-cell analyses. To address this limitation, we identify and resolve key memory and computational bottlenecks, and subsequently develop a GPU-accelerated version. Initially, we achieve a performance improvement in random forest training by incorporating the GPU-accelerated random forest implementation from *RAPIDS*. However, this approach remained hindered by the *CuPy* indexing issue. To overcome this challenge and support large and sparse input data, *ScaleSC* integrates the gradient boosted decision tree (XGBClassifier) from the *XGBoost* library (Chen and Guestrin 2016). *XGBoost* employs a specialized input format known as DMatrix, which is optimized for both speed and memory efficiency. Importantly, DMatrix is not affected by the *CuPy* indexing limitations, as it does not rely on *CuPy* for data handling. Our optimized approach enables the analysis of datasets with millions of cells while preserving *NSForest’*s strong feature selection performance. A comparison of results among *Scanpy*, *Seurat*, and *ScaleSC* is shown in Fig. 3.

### 2.5 Cluster reduction

Once cluster-specific marker genes have been identified, cell types can be assigned to clusters based on their respective marker gene expression. However, the clustering algorithms like the Leiden algorithm often produce a greater number of clusters than the cell types of interest, with many clusters sharing similar marker genes. This suggests that certain clusters can be further merged into larger groups. A potential approach to reducing the number of clusters is to rerun the clustering algorithm with a lower resolution. However, due to the algorithm’s inherent randomness, the clustering outcome varies across different runs. Consequently, users may prefer to reduce the number of clusters for cell type assignment while retaining the original clustering structure for other analyses. To address this issue, we implement a cluster-merging algorithm based on marker gene expression. This method provides users with the flexibility to consolidate clusters without altering their underlying structure. Formally, let *k* be the number of clusters, denoted as $C=\{C_{1},C_{2},\ldots ,C_{k}\}$. For each cluster $C_{i}$, the corresponding marker genes are represented as $G_{i}=\{g_{i}^{1},g_{i}^{2},\ldots ,g_{i}^{m}\}$. We define $S=\{S_{1},S_{2},\ldots ,S_{k}\}$ to quantify the association between marker genes and their respective clusters, these scores are provided by the decision tree F score, the normalized count matrix is denoted by *X*. First, we label clusters into two categories: high-quality and low-quality, based on a predefined threshold $t_{C}$. Next, we iterate over all cluster pairs $\left(C_{i},C_{j}\right)$ in *C* and count the number of marker genes in $G_{i}$ that are also markers of $C_{j}$. If this count exceeds a predefined threshold $t_{G}$, the pair $\left(C_{i},C_{j}\right)$ is added to the final candidate set *P*. Finally, cluster merging is performed exclusively for pairs $\left(C_{i},C_{j}\right)$ in *P*, where $C_{i}$ or $C_{j}$ is classified as a low-quality cluster. The complete algorithm is provided in Algorithm 1, available as supplementary data at *Bioinformatics Advances* online.

## 3 Results

### 3.1 Time efficiency

To comprehensively evaluate the performance of *Scanpy*, *Rapids-singlecell*, and *ScaleSC*, we selected datasets with three different scales: small, medium, and large. Each method has been tested across all datasets, from data loading to Leiden clustering, with the time taken for each step recorded for comparison. All tests were conducted on a node of a high-performance computing server (HPC) running Rocky Linux 8.10, equipped with 1 TB of CPU RAM and a single NVIDIA A100 GPU.

For the small dataset, we utilized the 70K human lung dataset (Travaglini *et al.* 2020), which contains 65 662 cells and 26 485 genes. All three methods were able to run on this dataset. As shown in Table 1, both *ScaleSC* and *Rapids-singlecell* exhibit exceptional performance, achieving a 15.4× speedup.

For the medium-scale dataset, we used the 1.3M mouse brain dataset (Zheng *et al.* 2017). *Rapids-singlecell* is not feasible for this dataset due to exceeding the *CuPy’*s int32 limitation. *ScaleSC* completed the task in 2 min, while *Scanpy* requires 4.5 h to process the entire dataset as shown in Table 2.

To assess the performance of *ScaleSC* on extremely large datasets, we generated a simulated dataset containing 13 million cells based on the original 1.3 million-cell mouse brain dataset. This expanded dataset was created by generating simulated samples from the original samples, with each sample being simulated nine times. For a given sample, new cells were generated for each cluster by modeling the distribution of gene expression within that cluster. The results, presented in Table 3, demonstrate that, with the optimizations implemented, *ScaleSC* completed the analysis in under 1 h while utilizing ∼60 GB of GPU memory. This performance indicates that *ScaleSC* is highly efficient for handling large-scale datasets and holds promise for scaling up to 20 million cells on a single A100 GPU. In contrast, both *Scanpy* and *Rapids-singlecell* failed to process such large datasets.

**Table 3.** The time cost of *ScaleSC* on a simulated data with over 13M cells and over 1000 samples.^a^

Step	*ScaleSC* (s)
Data loading	405
QC	686
Normalization	56
HVG	419
PCA	622
Harmony	628
Neighbors	163
UMAP	27
Leiden clustering	49
**All**	**3055**

a *Scanpy*, *Seurat*, and *Rapids-singlecell* are infeasible on this dataset.

A bold value is the sum of time cost for each algorithm.

### 3.2 Gene marker identification

We evaluated the performance of *ScaleSC* against *Scanpy* and *Seurat*(v5) (Hao *et al.* 2024b) on a public multiple sclerosis (MS) dataset (Absinta *et al.* 2021) comprising 66 432 cells with seven predefined cell types: Astrocytes, Immune, Lymphocytes, Neurons, Oligodendrocytes, OPC, and Vascular Cells. To assess baseline performance characteristics, the comparison was conducted without preliminary feature selection like highly variable gene filtering, which results in 25 449 candidate genes are being considered. *Rapids-singlecell* was excluded from this comparison due to its methodological similarity to *Scanpy’*s implementation.

The top five marker genes for each cell type are obtained from each method individually. For *Scanpy*, “scanpy.tl.rank_genes_groups” is used, while for *Seurat*, “FindMarkers” is applied. Both are called with default parameters. As shown in Fig. 3, the marker genes identified by *ScaleSC* demonstrated superior biological relevance, characterized by both increased cell-type specificity and elevated expression levels. Specifically, these markers exhibited higher group-wise cell fraction representation and enhanced expression intensity compared to traditional approaches like T-test, suggesting improved performance for cell-type discrimination.

### 3.3 Cluster merging

*ScaleSC* allows users to manually define a threshold $t_{G}$, which ranges between 0 and 1 with a default value of 1, as a more stringent merging criterion is encouraged. To evaluate the algorithm’s performance, we test it on the same MS dataset with seven cell types. This dataset has already been pre-clustered into 18 clusters using *Seurat*. These clusters and the identified markers in the previous section are subsequently used as inputs for *ScaleSC* to perform cluster merging. We evaluate the performance using two threshold values: 1.0 and 0.8.

**Figure 4.**
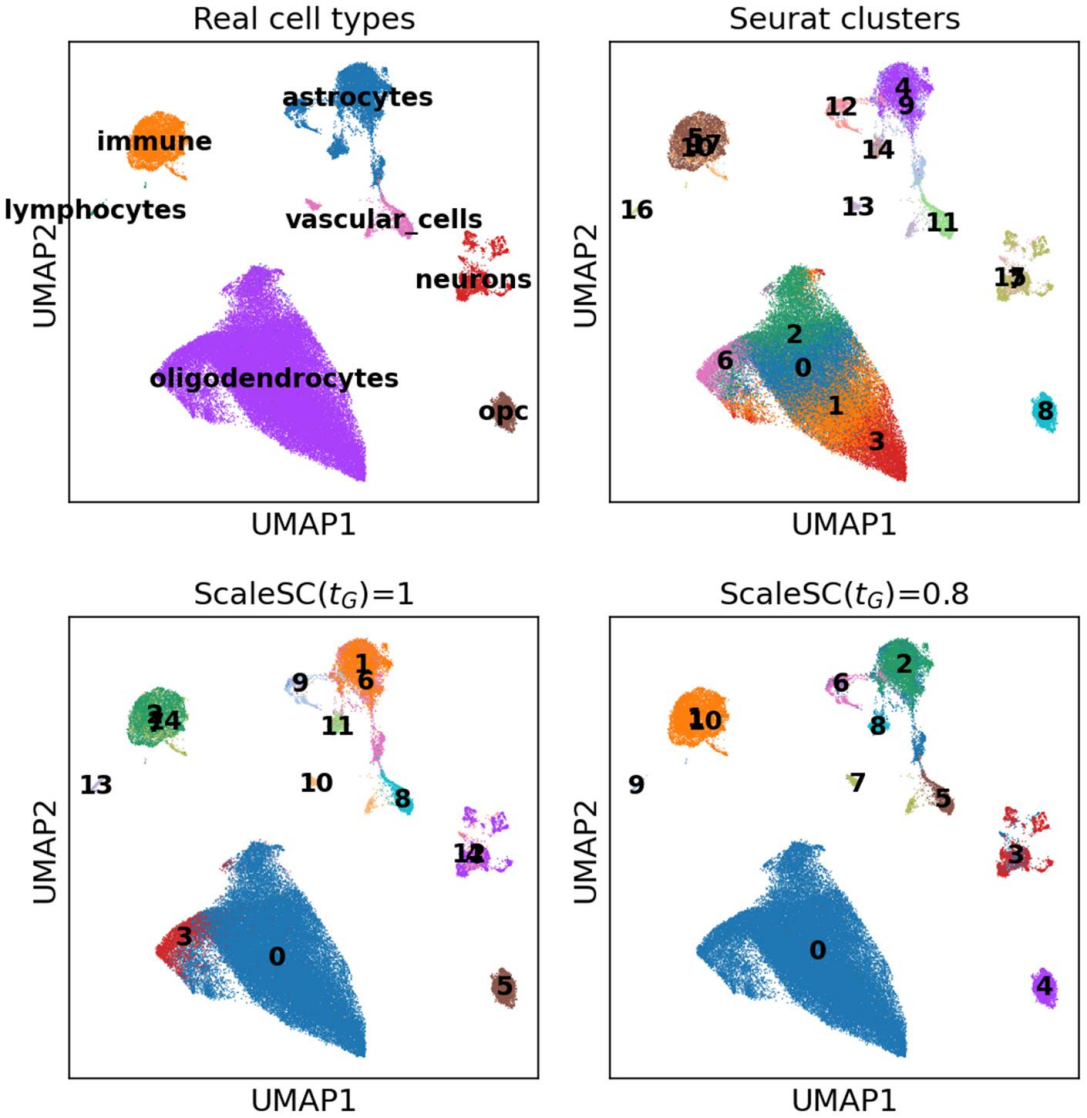
UMAP visualization of the performance of the cluster-merging algorithm with various $t_{G}$. upper left: real seven cell types; upper right: *Seurat* clusters, 18 clusters; lower left: $t_{G}$ is 1.0, 15 clusters; lower right: $t_{G}$ is 0.8, 11 clusters. Time: 40.2 s.

As shown in Fig. 4, a threshold of $t_{G}=1$ already demonstrates relatively strong merging performance. For instance, *Seurat* clusters 0, 1, 2, and 3 are merged into a larger cluster corresponding to the Oligodendrocytes cell type, while cluster 6 remains unmerged. When the threshold is lowered to $t_{G}=0.8$, *Seurat* cluster 6 is further merged and then labeled as Oligodendrocytes. Another example using the 1.3 million mouse brain dataset with varying thresholds is provided as Fig. 1, available as supplementary data at *Bioinformatics Advances* online, and the process can be completed within 4 min for both marker finding and cluster merging.

## 4 Conclusion

The growing scale of single-cell RNA-seq data presents significant challenges in terms of processing speed and memory capacity. In this work, we introduced *ScaleSC*, a GPU-accelerated solution designed specifically to address these challenges for users with limited computing resources, such as those with a single GPU or without deep familiarity with GPU ecosystems. By building upon the powerful foundations of *CuPy*, *Scanpy*, and *Rapids-singlecell*, *ScaleSC* delivers over a 20× speedup in processing, overcoming memory bottlenecks and enabling the analysis of datasets with 10–20 million cells and over 1000 batches on a single A100 GPU. In addition to improving scalability, *ScaleSC* ensures consistent results by resolving discrepancies between GPU and CPU algorithm implementations. The package also includes GPU-optimized tools for advanced tasks like marker gene identification and cluster merging, providing more efficient and better solutions for marker identification and clusters refinement. Designed for ease of use, *ScaleSC* integrates seamlessly into existing *Scanpy* and *Rapids-singlecell* workflows, requiring minimal adaptation from users. With its substantial improvements in speed, scalability, and accessibility, *ScaleSC* offers significant benefits to the single-cell RNA-seq community, particularly for those with limited computational resources, providing an accessible and high-performance tool for large-scale data analysis.

## Supplementary Material

vbaf167_Supplementary_Data

## Data Availability

The datasets used for benchmarking runtime are as follows: The 70k human lung dataset is available at Synapse (https://www.synapse.org/Synapse: syn21041850/wiki/600865). The 1.3M mouse brain dataset is provided by NVIDIA and is available at https://github.com/NVIDIA-Genomics-Research/rapids-single-cell-examples/blob/master/notebooks/1M_brain_cpu_analysis.ipynb, or downloaded from the 10× Genomics platform. The 13M simulated dataset is available upon request, as its size exceeds typical storage limits. The single-cell transcriptomic data used for benchmarking marker gene identification and clustering are publicly available in the Gene Expression Omnibus (GEO) under accession number GSE180759.
