# Supplementary material for: ScaleSC: a superfast and scalable single-cell RNA-seq data analysis pipeline powered by GPU
Source: Bioinform Adv. 2025 Jul 17;5(1):vbaf167. doi: 10.1093/bioadv/vbaf167 (PMC12321287; doi:10.1093/bioadv/vbaf167)
Supplement: vbaf167_Supplementary_Data [file vbaf167_supplementary_data.pdf]

ScaleSC: A superfast and scalable single cell RNA-seq data analysis pipeline powered by GPU
Supplementary Material

Algorithm 1 An algorithm for clustering merging

```
Data: X, C, G_i, S, t_C, t_G
Result: P
C → {C_high, C_low} ;                                   /* split C into two parts */
C_high = {C_i} if S_i ≥ t_c ;                          /* high quality part */
C_low = {C_i} if S_i < t_c ;                           /* low quality part */
P = ∅ ;                                                 /* init P as an empty set */
for C_i in C do
    ;                                                   /* enumerate pairwise clusters */
    for C_j in C do
        n = 0
        for g in G_i ;                                  /* enumerate each marker of C_i */
        do
            if mean(X^g_{C_i}) − std(X^g_{C_i}) ≤ mean(X^g_{C_j}) then
                n = n + 1 ;                             /* the number of shared markers */
            end
        end
        if n/|G_i| ≥ t_G then
            P ← (C_i, C_j) ;                            /* add the pair into P */
        end
    end
end
for (C_i, C_j) in P do
    if C_i in C_low or C_j in C_low ;   /* only merge the pair with at least one cluster in C_low */
    then
        P ← (C_i, C_j)
    end
end
merge all pairs in P
```

Table S1. Some large variables produced during calculation (13 million cells), d denotes the dimension of PC loadings, B denotes the number of samples, C denotes the number of clusters (up to 100).

Variable	Description	Shape	Size
Z_orig	PCA input	(d, N)	$2.5G$
Z_cos	L2 normalization of input, cosine distances	(d, N)	$2.5G$
Z_corr	Corrected embeddings	(d, N)	$2.5G$
Phi	The one-hot encoded batch assignment matrix.	(B, N)	$64G$
Phi_moe	The one-hot batch assignment with bias in MoE estimation	$(B+1, N)$	$64G$
Phi_Rk	The one-hot batch assignment for a given cluster	$(B+1, N)$	$64G$
R	the cluster assignment matrix	(C, N)	$5G$
dist_mat	Distance matrix	(C, N)	$5G$
scale_dist	Scaled distance matrix	(C, N)	$5G$

we test it on the previous 1.3M mouse brain dataset filtered by highly variable genes(4,000) with clusters inferred from the Leiden algorithm using three different thresholds: 0.9, 0.8, and 0.7. As expected, a lower threshold generally results in fewer clusters, as more clusters are merged based on marker gene similarity. It takes 220 seconds to finish both marker finding and cluster merging on a single A100.

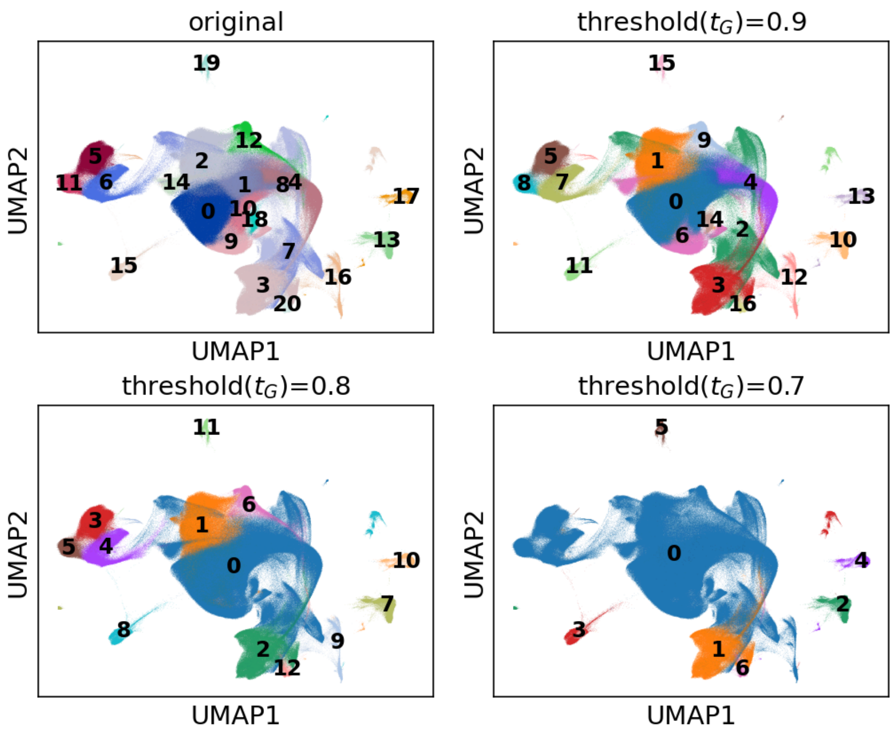

Fig. S1. UMAP visualization of the performance of the cluster-merging algorithm with various t_G. upper left: the original 21 clusters; upper right: t_G is 0.9, 17 clusters; lower left: t_G is 0.8, 13 clusters; lower right: t_G is 0.7, 7 clusters.

Table S2. The difference between ScaleSC and Rapids-singlecell in each step, from beginning to the end, in order.

Feature	Implementation
**Input**	Both Scanpy and Rapids-singlecell require a single H5AD or H5 file containing the entire dataset, whereas ScaleSC expects a directory containing multiple H5AD or H5 files, with each file corresponding to an individual sample.
**Memory Management**	Rapids-singlecell relies on the RMM package to manage memory across GPU and CPU, whereas ScaleSC handles memory management and communication internally using its own built-in mechanisms. ScaleSC supports three data-loading modes (GPU-only, CPU-only, Disk-only), providing flexibility based on available resources. Distributed storage across multiple GPUs is supported, though computations are performed on a single GPU.
**Quality Control (QC) & Filtering**	ScaleSC uses chunk-based implementation for QC and filtering.
**Normalization**	ScaleSC implements chunk-based normalization and log1p transformation using `rsc.pp.normalize_total` and `rsc.pp.log1p`.
**Highly Variable Genes (HVG)**	ScaleSC uses chunk-based implementation of the `seurat_v3` algorithm.
**PCA**	ScaleSC uses Chunk-based PCA for scalability. ScaleSC corrects the sign flipping of PC loadings based on `sign(max(abs(eigenvalues)))` to ensure the consistency with Scanpy's results.
**Harmony**	ScaleSC uses a memory-efficient implementation of Harmony, enabling scaling up to 20M cells and 1000 samples.
**Neighbors**	Same as `rsc.pp.neighbors`.
**Leiden**	Same algorithm as `rsc.tl.leiden`. The cluster output of ScalseSC is sorted based on cluster size, whereas Rapids-singlecell doesn't apply any ranking.
**UMAP**	Same as `rsc.tl.umap`.
**Marker Identification**	ScaleSC uses a novel method, mainly based on NS-Forest, for gene marker identification, whereas Scanpy and Rapids-singlecell use conventional methods, e.g., t-test.
**Cluster Merging**	A novel marker-based cluster merging algorithm is used in ScaleSC to reduce cluster granularity.
